# EFFECTS OF POSTURAL EDUCATION IN ELEMENTARY SCHOOL CHILDREN: A SYSTEMATIC REVIEW

**DOI:** 10.1590/1984-0462/2021/39/2020005

**Published:** 2020-10-28

**Authors:** Paola Janeiro Valenciano, Fabíola Unbehaun Cibinello, Jessica Caroliny de Jesus Neves, Dirce Shizuko Fujisawa

**Affiliations:** a Universidade Estadual de Londrina, Londrina, PR, Brazil.

**Keywords:** Child, Health education, Posture, Criança, Educação em saúde, Postura

## Abstract

**Objective:**

To determine the effect of postural education on the learning and postural habits of elementary school children without physical intervention.

**Methods:**

We searched PubMed, Lilacs, SciELO, Cochrane, and Science Direct data bases and reference lists of studies in February 2020. The eligibility criteria were randomized clinical trials related to the effect of postural education in children aged between 6 and 12 years old. Two authors independently assessed trials for inclusion and risk of bias: randomization process, deviations from intended interventions, missing outcome data, measurement of the outcome, and selection of the reported result. Data were extracted in standardized tables including information on author, publication year, country, sample size, age, sex, intervention characteristics, outcome measurements and results.

**Results:**

We found seven clinical trials (involving 2,568 children) for the review. The studies were conducted between 2000 and 2018: four in Belgium, two in Spain, and one in Germany. All seven included trials underwent evaluation: only one had a clear process of randomization and allocation concealment. All included studies were judged as having high risk of bias in at least one domain or have concerns for multiple domains.

**Conclusions:**

The positive effects of acquired knowledge and postural habits found in the studies cannot be used to reliably support postural education in elementary school children due to a high risk of bias in the evaluated studies.

## RESUMO

**Objetivo:**

Determinar os efeitos da educação postural na aprendizagem e nos hábitos posturais de crianças do ensino fundamental sem intervenção física.

**Métodos:**

Foram realizadas buscas nas bases de dados do PubMed, Lilacs, SciELO, Cochrane e Science Direct e nas listas de referência dos estudos em fevereiro de 2020. Os critérios de elegibilidade foram ensaios clínicos randomizados relacionados aos efeitos da educação postural em crianças de 6 a 12 anos de idade. Duas autoras avaliaram os ensaios de forma independente para inclusão e risco de viés: processo de randomização, desvios das intervenções pretendidas, ausência de dados do desfecho, mensuração do desfecho e seleção do resultado relatado. Os dados foram extraídos em tabelas padronizadas e incluíram informações sobre o autor, ano de publicação, país, tamanho da amostra, idade, sexo, características da intervenção, mensuração do desfecho e resultados.

**Resultados:**

Foram encontrados sete ensaios clínicos (envolvendo 2.568 crianças) para a revisão. Os estudos foram realizados entre 2000 e 2018: quatro na Bélgica, dois na Espanha e um na Alemanha. Todos os sete estudos incluídos foram submetidos à avaliação e apenas um apresentou um processo claro de randomização e ocultação de alocação. Todos os ensaios foram considerados como de alto risco de viés em pelo menos um domínio ou preocupantes em vários domínios.

**Conclusões:**

Os efeitos positivos encontrados relacionados ao conhecimento adquirido e aos hábitos posturais não podem ser utilizados para recomendar de forma confiável a educação postural para escolares do ensino fundamental, devido ao alto risco de viés dos estudos avaliados.

## INTRODUCTION

During development, children acquire postural habits that they tend to adhere to for the rest of their lives.^1^ According to Noll et al.,^2^ most students use an inadequate posture to carry out activities such as writing, using the computer, and picking up objects from the ground. Furthermore, children and adolescents frequently suffer from musculoskeletal pain, particularly in the back and neck,^3^ but can learn healthy habits which could prevent future pain.^4^

Zapater et al.^5^ highlighted the higher efficiency of preventive approaches to musculoskeletal problems when the child is in a growth phase and propose further research on educational programs on seated posture in the classroom so that this issue may be effectively addressed. Grors et al.^6^ raised important reflections on the incidence of spinal column pain in the population as well as necessary strategies to achieve genuine social change. They also argued that educational initiatives should be directed toward individuals in their formative age, a phase in which attitudes and beliefs are being shaped. They also discussed how strategies, such as public education, social marketing, and intervention policies should be aimed at the child population.

The classroom is among the diverse contributing factors to the manifestation of musculoskeletal symptoms in school children.^7^ For instance, the use of school bags, a common practice in elementary school children is a risk factor for musculoskeletal discomfort.^8^ Marques et al.^9^ discussed the prolonged time children spend sitting, which is a risk factor for lumbar pain. Furthermore, different studies have revealed the existence of shortcomings in the anthropometric measurements of service users and the furniture used in schools.^9^ ^,^ ^10^ As a result, studies have advanced toward detecting the impact of poor posture, both in relation to pain and to postural deficiencies, and as a barrier to concentration and learning.^9^ ^,^ ^10^ ^,^ ^11^

Health and education professionals play an important role in schools, and given the known risk of children developing inappropriate behaviors and postures as time passes, these can entail a functional compromise.^12^ Whereas this is a relatively current issue,^13^ the short-, medium-, and long-term effects of postural education strategies for elementary school children are not clear. Thus, the present systematic review aims to evaluate postural education effects relating to acquired knowledge and postural habits in children from 6 to 12 years old.

## METHOD

The study was based on the guidelines of the Preferred Reporting Items for Systematic Reviews*-* PRISMA, but there is no protocol registration.^14^

**Chart 1:**
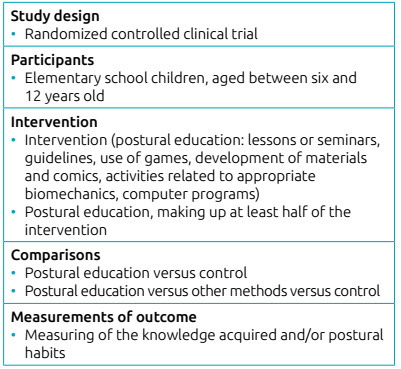
Eligibility criteria.

This study included original articles on clinical trials relevant to the effect of postural education in children between 6 and 12 years old (Chart 1). Exclusion criteria were recommendation studies, incomplete texts, duplicated articles, study protocols, pilot studies, and studies classified as quasi-experimental.

In February 2020, we systematically searched five databases: PubMed, Latin American and Caribbean Health Sciences Literature (Lilacs), Scientific Electronic Library Online (SciELO), Cochrane Central Register of Controlled Trials (CENTRAL), and Science Direct. Search terms included (child* OR students OR pediatr*) AND (postu* OR spine OR spinal curvatures) AND (health promotion OR school health services OR educ* OR quality of life) AND (trial). We set no limitations as to language or publication date.

**Figure 1:**
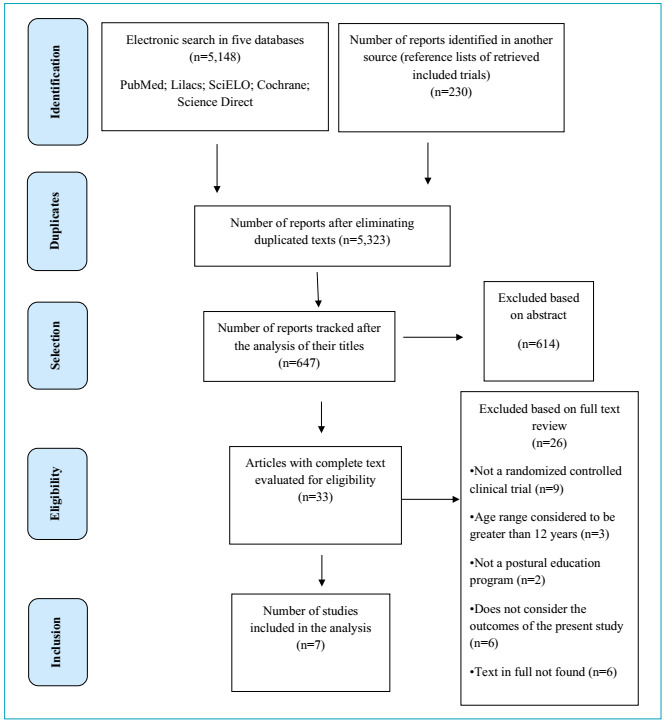
PRISMA 2009 flowchart.

Two authors (PJV and FUC) independently reviewed the titles and abstracts of the identified articles. Subsequently, the complete texts of potentially relevant studies were analyzed, and any disagreements were resolved by a third examiner (JCJN). In addition, we attempted to identify other potentially eligible trials by searching the reference lists of the retrieved included trials (other source). No contact was made with study authors to identify additional studies. The number of articles in each screening stage is shown in Figure 1. EndNote X8.2 was used to manage bibliographic references and visualize duplicated references.

**Figure 2:**
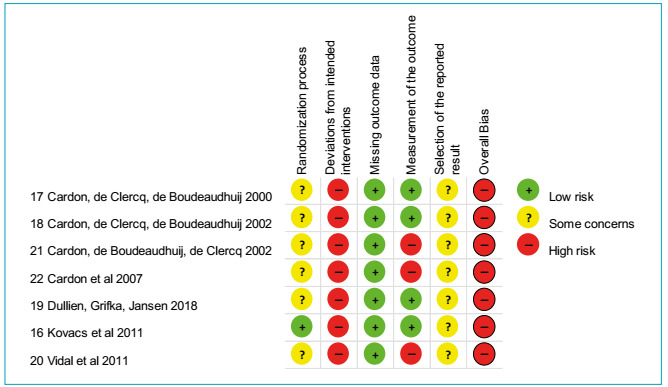
Evaluation of the risk of bias of the studies included using the RoB 2 tool from Cochrane.^15^

Two reviewers (PJV and FUC) independently evaluated the risk of bias and, if necessary, consulted a third review author (JCJN) for all included studies, in accordance with the recommendations by the Cochrane Collaboration, which recommends using version 2 of the Cochrane risk-of-bias tool for randomized trials (RoB 2).^15^ The following items were evaluated: randomization, allocation concealment, blinding of the participant and researchers, blinding of the evaluation, incomplete data, selective publication and other biases (Figure 2).

Data extraction and summary were undertaken according to author, publication year, country, sample size, age, sex, intervention characteristics, outcome measurements and results. More detailed data on intervention were collected, such as the professionals involved, the composition of the postural education program, and additional interventions.

For all continuous variables, we extracted sample sizes, means and standard deviations for each intervention and control group. The data were inserted into a spreadsheet in Excel program. However, since the meta-analysis was not possible due to methodological heterogeneity, the data were organized in a form, and we reported findings descriptively.

## RESULTS

Of the total of 5,378 studies, 55 were excluded because of duplication, 4,676 were excluded based on the analysis of their titles and 614 were excluded based on abstracts. Of the 33 selected for complete text analysis, 26 were excluded due to the eligibility criteria. As a result, seven studies were chosen for systematic review (Figure 1).

Regarding the risk of bias, each trial was rated as high risk, unclear risk or low risk on the following domains: 1-randomization process, 2-deviations from intended interventions, 3-missing outcome data, 4-measurement of the outcome, and 5-selection of the reported result. The RoB 2 tool by Cochrane includes overall risk-of-bias judgement. Whereas all the studies had low risk of bias for missing outcome data (domain 3),^16^ ^,^ ^17^ ^,^ ^18^ ^,^ ^19^ ^,^ ^20^ ^,^ ^21^ ^,^ ^22^ and four studies had low risk of bias for measurement of the outcome (domain 4),^16^ ^,^ ^17^ ^,^ ^18^ ^,^ ^19^ only one^16^ had a clear process of randomization and allocation concealment clear (domain 1) and, in addition, all included studies were judged as high risk of bias in at least one domain for their results or were presented some concerns for multiple domains in a way that substantially lowers confidence as to their the result. Figure 2 shows the scores for these studies.

The characterization of the included studies is shown in Table 1. A total of 2,568 elementary school children participated in the clinical trials. The studies were undertaken between 2000 and 2018: four in Belgium,^17^ ^,^ ^18^ ^,^ ^21^ ^,^ ^22^ two in Spain,^16^ ^,^ ^20^ and one in Germany.^19^

**Table 1:** Characteristics of the included studies.

Study/Country	Participants	Intervention
Cardon et al.^17^/Belgium	n=78 Age (years)=9.93 in EG and 11.10 in CG Sex=35 M, 43 F	CG=No intervention; EG=Postural education, exercises taught to the children, sessions, and provision of materials for parents and teachers Basis of the educational program: Literature on biomechanics and the German Back School Duration: Six 60-minute sessions, with a one-week interval between sessions; Follow-up: Three months
Cardon et al.^18^/Belgium	n=363 Age (years)=9.8 in EG and 10.3 in CG Sex=171M, 192 F	CG=No intervention; EG=Postural education; information session for parents and teachers, and teachers requested to be present in all sessions Basis of the educational program: Literature on biomechanics and the German Back School Duration: Six 60-minute sessions, with one-week intervals between sessions; Follow-up: One year.
Cardon et al.^21^/Belgium	n=706 Age (years)=10 (±0.6 in EG and ±0.7 in CG) Sex=401 M, 305 F	CG=No intervention; EG=Postural education, sessions, and provision of materials for parents and teachers, teachers present in all sessions Basis of educational program: Previous studies^17^ Duration: Six 60-minute sessions, with one-week intervals between sessions; Follow-up: Three months and one year
Cardon et al.^22^/Belgium	n=603 Age (years)=9.7±0.7 Sex=289 M, 314 F	CG=No intervention; EG=Postural education; EG+PA=Postural education and physical activity program Basis of the educational program: Previous studies^18^ ^,^ ^21^ Duration: Six sessions with one-week intervals between sessions; Follow-up: None
Dullien et al.^19^/Germany	n=176 Age (years)=10.5±0.4 Sex=76M, 100 F	CG=No intervention; EG=Five lessons on back care (provided material), posture awareness training and improvement in the classroom, and back and abdominal muscle exercises at the beginning of each lesson Duration: One year; Follow-up: None
Kovacs et al.^16^/Spain	n=497 Age (years)=8 Sex=260M, 237 F	CG=No intervention; EG=The professor was advised only to relay the comic story about the spine to each student Basis of the educational program: “Back Book” Duration: One session; Follow-up: Three months
Vidal et al^20^/Spain	n=145 Age (years)=10.72±0.672 Sex=52.8% M, 48.2% F	CG=No intervention; EG=Four theoretical educational sessions and two practical ones Basis of the educational program^22^ ^,^ ^23^ ^,^ ^24^ ^,^ ^25^ ^,^ ^26^ Duration: Six sessions; Follow-up: Three months

EG: experimental group; CG: control group; PA: physical activity; SD: standard deviation; M: male; F: female; n: sample number; min.: minutes.

Acquired knowledge was evaluated in five studies,^16^ ^,^ ^17^ ^,^ ^19^ ^,^ ^21^ ^,^ ^22^ all using questionnaires to measure changes related to this outcome, although no questionnaires were the same (Table 2). Dullien et al.^19^ showed a significant short-term improvement in acquired knowledge with postural education. Two studies^16^ ^,^ ^17^ found a significant increase in acquired knowledge by the experimental group both in the short- and medium-term. Cardon et al.^21^ showed a significant short-, medium-, and long-term improvement in acquired knowledge by the experimental group, along with an increase in the percentage of correct answers regarding general and specific knowledge in the same group one year post intervention. Cardon et al.^22^ found that after postural education, both the postural education group and the group associated with a physical activity program significantly increased their knowledge regarding the care for the spinal column in the short term, with no significant differences between them (Table 3).

**Table 2:** Measurements of outcome of the included studies.

Study	Measurements of outcome
Cardon et al.^17^	• Knowledge acquired: -Questionnaire testing knowledge related to the spinal column 13 multiple-choice items • Postural habits: -Individual practical test, filmed: choice of most appropriate furniture, sitting down, standing up from the ground, picking up a pen from the ground, carrying school bag, writing; resources such as the telephone book could be used; score from 0 (very poor) to 4 (excellent)
Cardon et al.^18^	• Postural habits: -Practical test: movement session with different tasks: removing shoes, sitting down, dealing with and moving a box, picking up a small object and using a schoolbag; the better the body biomechanics, the better the score (each test varied the score from 0 to 4) -Evaluation of postural habits with a hidden camera: observation in the classroom and in the movement session with tasks undertaken in groups of two with activities, such as throwing a ball to each other
Cardon et al.^21^	• Knowledge acquired: -Questionnaire with 12 multiple-choice items on general knowledge about spinal care, 10 items on specific knowledge • Postural habits: -Questionnaire with four items on self-reported behavior
Cardon et al.^22^	• Knowledge acquired: -Questionnaire on knowledge about spinal care with 11 items (based on previous studies)^18^ ^,^ ^21^ • Postural habits: -Observation of behaviors in spinal care during the movement session through filming, based on the study by Cardon et al.^18^
Dullien et al.^19^	• Knowledge acquired: -Questionnaire with 12 questions related to five back-care lessons (total=24 points) • Postural habits: -Tasks: lifting, carrying, balancing on a marked line, correct turning, and putting down a mineral water crate (0-2 points could be achieved) *Midterm evaluation=After four months
Kovacs et al.^16^	• Knowledge acquired: - Questionnaire with10 statements focusing on ways to prevent or manage pain in the back (true or false)
Vidal et al.^20^	• Postural habits: -Questionnaire on daily postural habits with seven items (only six items used for analysis) on daily living habits: scored as 0=no and 1=yes

EG: experimental group; CG: control group; PA: physical activity; SD: standard deviation; M: male; F: female; min.: minutes.

**Table 3:** Results of the included studies.

Study	Results
Cardon et al.^17^	• ↑Knowledge acquired in the immediate post-test and follow-up (p<0.001) • Postural habits: -Better scores in the EG in the immediate post-test period and follow-up (p<0.001)
Cardon et al.^18^	• Postural habits: -EG presented a higher score after the intervention, after three months and one year for all the items and total score -The increase in the total score for the practical test pre intervention evaluation and evaluation after a one-year follow-up was +1.14 for CG and +26.5 for EG -In the evaluation with the hidden camera, the score was significantly greater in EG (p<0.001) one year after the intervention
Cardon et al.^21^	• Knowledge acquired: -↑Knowledge acquired in the immediate post-test period and follow-up of three months and one year (p<0.001) -The improvement in general knowledge in the immediate post-test at one year was 33% in EG and 12% in CG; for specific knowledge, 21% in EG and 6% in CG • Postural habits: -Self-reporting of checking schoolbag weight: EG scored higher in the pre- and all post-tests (p<0.001). -Posture when taking the shoes off: EG scored significantly higher in the post-test at three months, whereas posture when picking things up and carrying them was significantly higher in the immediate post-test and at three months
Cardon et al^22^	• Knowledge acquired: -↑Knowledge acquired regarding spinal care in EG and EG+PA (p<0.001); No significant difference between EG and EG+PA • Postural habits: -Total score for spinal care behaviors was significantly greater in the EG than in CG (p<0.001) and greater in EG than in EG+PA (p<0.001)
Dullien et al.^19^	• Knowledge acquired: -EG significantly improved their knowledge; there was a significant interaction between “group” and “test time” (F (1.123)=11.87, p=0.001) • Postural habits: -EG improved their behavior in the water crate-carrying task; there was a significant interaction between the factors “group” and “test time” (F (1.164)=7.93, p=0.005)
Kovacs et al^16^	• Knowledge acquired: -↑Knowledge acquired with the intervention and the effect continued to be significant after three months (p<0.001) -The success in EG, when compared to CG was 1.61 times greater (CI95%: 1.03-2.52, p=0.038)
Vidal et al^20^	• Postural habits: -↑in score for healthy habits in the post-test in comparison with the baseline in EG (p<0.001) and maintained after three months of follow-up (p < 0.001) -No significant changes observed in CG (p>0.6)

EG: experimental group; CG: control group; PA: physical activity.

Postural habits were investigated in six studies: four in practical tests/filmed-movement sessions,^17^ ^,^ ^18^ ^,^ ^19^ ^,^ ^22^ and two with questionnaires (Table 2).^20^ ^,^ ^21^ In the study by Cardon et al.,^17^ the filming was individual, and significant short- and medium-term improvements were found as to the intervention. Cardon et al.^18^ also evaluated postural habits using a hidden camera and tasks performed in pairs and showed a significant effect of the intervention with an improvement in scores in the medium and long term. Meanwhile, Dullien et al.,^19^ using task observation, found that only the experimental group improved their behavior in the water crate-carrying task. Cardon et al.^22^ used filming, based on the study by Cardon et al.,^18^ and found that the group that received postural education and postural education associated with a physical activity program presented significant behavioral improvements related to spinal care than the control groups. They also found that the group that underwent postural education had a significantly higher score than the group that received postural education associated with a physical activity program in the short term. Cardon et al.^21^ evaluation of a questionnaire with items on self-reported behavior showed that the postural education group presented a significant increase in the frequency of checking school bag weight in the medium and long term, in their posture when taking off shoes in the medium term, and in picking things up and carrying them in the short and medium term. Meanwhile, Vidal et al.^20^ used a questionnaire on daily postural habits and showed a significant improvement in scores for healthy habits in the short and medium term (Table 3).

Regarding postural education, the interventions varied from one to six sessions. Five studies used six sessions for postural education, of which four reported a one-week interval between sessions and only three reported 60 minutes for each session. There was a lack of information on number and duration of sessions in only one study.^19^ In the Belgium studies,^17^ ^,^ ^18^ ^,^ ^21^ ^,^ ^22^ the interventions were conducted by physiotherapists. Cardon et al.^22^ presented an additional intervention for postural education that included two intervention groups: postural education and postural education associated with promoting physical activity. Meanwhile, Dullien et al.^19^ included static and dynamic exercises associated with postural education. The detailed characteristics of the interventions related to postural education are shown in Table 4. As the theoretical basis for structuring educational programs, the authors relied on the literature,^22^ ^,^ ^23^ ^,^ ^24^ ^,^ ^25^ ^,^ ^26^ studies on biomechanics,^27^ the German Back School,^28^ ^,^ ^29^ previous studies by the same authors,^17^ ^,^ ^18^ ^,^ ^21^ ^,^ ^30^ and referent literature to the “Back Book”,^22^ ^,^ ^33^ as well as the cooperation of Orthopedic residents, psychologists, sports scientists, and teachers (Table 1).^19^

**Table 4:** Characteristics of the postural education sessions.

Study	Professional involved in the postural education	Guided discovery and active methodology	Games, movements and exercises based on daily activities	Ten guidelines on “how to make your disks happy”	Comic book about the spine	Characters: “Fit Fred” and “Lazy Leo”	Additional strategies	Additional interventions
Cardon et al.^17^	Physiotherapist	Yes	No	Yes	Yes	Yes	Information and materials provided to parents, children, and teachers; teachers present in the sessions	No
Cardon et al.^18^	Physiotherapist	Yes	No	Yes	Yes	No	Information session for parents and teachers; teachers present in the sessions	No
Cardon et al.^21^	Physiotherapist	Yes	No	Yes	Yes	No	Information and materials provided to parents and teachers; teachers received extra exercises to be used in the classroom	No
Cardon et al.^22^	Physiotherapist	No	No	No	Yes	Yes	Guidelines provided for teachers to integrate the principles into lessons	Balls, a Dynair, a sitting wedge, and lessons for developing and maintaining an active lifestyle; extracurricular sports session were provided
Dullien et al.^19^	Teacher	Yes	Yes	No	No	No	Posture awareness training; healthy lifting and carrying, back-friendly sports, and the importance of reducing sitting behavior were explained	Static exercises to be completed three times (each position held for 15-20s), as well as dynamic exercises (each with 15-20 repetitions)
Kovacs et al.^16^	Teacher	No	Yes	No	No	No	No	No
Vidal et al.^20^	Not clear	No	No	No	No	No	Two practical sessions: postural analysis, carrying objects, balance, breathing, and relaxation	No

## DISCUSSION

Health and education services that align, integrate, and collaborate in partnership can improve efficiency, reduce resource consumption, and produce better results.^34^ According to the Centers for Disease Control and Prevention (CDC),^35^ establishing healthy behaviors in children is more advantageous and easier than trying to change already-established unhealthy habits in adulthood. In this regard, the schools perform a fundamental role.

The present review identified interventions with various components adapted to children’s age range, and which were tested in randomized trials as options for providing postural education to elementary school children. After analyzing the postural education sessions, all the proposals were adapted to the child population, including active methodology, games, comic books, and characters, among others, and worked on the concepts of biomechanics, the spinal column, and posture. In this regard, Jachyra and Fusco^36^ discussed the potential benefits of schools’ implementation of play-based learning to children’s health and well-being, given that playing is a fundamental right of children and an opportunity for them to be active. In addition, three studies included an information session for parents and teachers. Cardon, de Bourdeaudhuij, and de Clercq^21^ emphasized the premise that parents have a fundamental role in shaping their children’s health choices.

Lewallen et al.,^37^ discussing the role of health education for students, provided by qualified and trained teachers, emphasized that health education helps students acquire knowledge, attitudes, and skills necessary for adopting health-enhancing behaviors and for becoming agents of health promotion in their communities. The authors also highlighted that the initiatives and collaborative actions of health professionals, such as nurses, dentists, and physicians are important in addressing school children’s actual and potential health problems. In the present review, most professionals involved were physiotherapists who, in the body of the profession’s knowledge, undertake in-depth studies into Anatomy, Pathology, and Biomechanics, including a deep and broad understanding of normal movement and impaired function. As a result, these professionals are critical agents in the promotion of health and well-being, who educate individuals and their family members on managing their health conditions to maximize their quality of life.^38^

Future concerns remain, such as those related to children’s increasing use of computers, but the ergonomic guidelines remain below adult standards,^39^ as shown in the study by Howie et al.,^40^ which found that to minimize potential musculoskeletal and sedentary lifestyle risks, playing with “non-screen” toys should be encouraged, along with education and advice provided to parents and caregivers. Balkó et al.^41^ discussed the increase in studies showing a rising trend toward a sedentary lifestyle in elementary schoolchildren and proposed, as a preventive measure, an increase in physical education classes in schools or interaction between state institutions, schools, families, and sports clubs to improve the amount of children’s daily activity.

For the main results, the positive effects as to acquiring knowledge and postural habits found in the studies cannot be used to reliably support postural education in elementary school children. The findings were limited by the high risk of bias in the evaluated studies, and the heterogeneity in the research methodologies did not allow meta-analysis of the results. Checking reference lists of included trials was undertaken to minimize the potential source of bias of the search strategy, which may not have retrieved all relevant papers. Another limitation is that there is no protocol registration in the PROSPERO registration record.

Evidence available at the time of writing cannot be used to reliably support postural education in elementary school children, thus reinforcing the importance of researching postural education for school children’s health and the role played by professionals in its promotion.
